# Evolving Landscape of Sickle Cell Anemia Management in Africa: A Critical Review

**DOI:** 10.3390/tropicalmed9120292

**Published:** 2024-11-29

**Authors:** Hazel W. Musuka, Patrick Gad Iradukunda, Oscar Mano, Eric Saramba, Pierre Gashema, Enos Moyo, Tafadzwa Dzinamarira

**Affiliations:** 1 School of Medicine, Medical Science and Nutrition, University of Aberdeen, Aberdeen AB25 2ZR, UK; 2 Rwanda Food and Drug Authority, Kigali 3243, Rwanda; gadpatrickiradukunda@gmail.com; 3 Department of Medical Laboratory Sciences, University of Zimbabwe, Harare 263, Zimbabwe; 4 College of Medicine and Health Sciences, University of Rwanda, Kigali 3286, Rwanda; 5 School of Nursing and Public Health, Faculty of Medicine and Health Sciences, University of KwaZulu Natal, Durban 4000, South Africa; 6 School of Health Systems & Public Health, University of Pretoria, Pretoria 0028, South Africa; 7 ICAP in Zimbabwe, Harare 263, Zimbabwe

**Keywords:** sickle cell disease, Africa, disease management

## Abstract

Sickle cell disease (SCD) is a prevalent inherited blood disorder, particularly affecting populations in Africa. This review examined the disease’s burden, its diverse clinical presentations, and the challenges associated with its management in African settings. Africa bears a significant burden of SCD, with prevalence varying across countries and age groups. Newborn screening programs have highlighted the high prevalence of SCD at birth, emphasizing the need for early diagnosis and intervention. The clinical manifestations of SCD in Africa are multifaceted, encompassing acute complications like vaso-occlusive crises, acute chest syndrome, and stroke, as well as chronic complications such as organ damage and leg ulcers. Biological factors, including fetal hemoglobin levels, and demographic factors, like age and sex, influence disease severity and outcomes. The management of SCD in Africa faces numerous challenges. Limited access to resources, including diagnostic tools, medications, and trained healthcare professionals, hinders optimal care. The high cost of advanced therapies further restricts patient access. Cultural stigma and a lack of awareness create additional barriers to effective management. To address these challenges, early diagnosis through newborn screening programs and point-of-care testing is crucial. Comprehensive care models, including hydroxyurea therapy, pain management, and patient education, are essential for improving outcomes. Collaboration with international networks and leveraging local resources can enhance the sustainability of SCD programs. In conclusion, SCD significantly impacts African populations. Overcoming the challenges associated with its management requires addressing resource limitations, affordability issues, and cultural barriers. Early diagnosis, comprehensive care models, and ongoing research focused on affordability and accessibility are crucial for improving the lives of individuals living with SCD in Africa.

## 1. Introduction

Sickle cell disease (SCD) is the most prevalent inherited blood disorder, affecting approximately 5% of the global population [1,2,3,4]. The epidemiology of SCD is closely linked to the sickle cell trait (SCT) prevalence [5]. SCT refers to the heterozygous condition where an individual inherits one normal hemoglobin gene (HbA) and one sickle hemoglobin gene (HbS). People with SCT typically do not exhibit symptoms of sickle cell disease (SCD) but can pass the sickle hemoglobin gene to their offspring. Sickle thalassemia refers to a condition where an individual inherits one sickle hemoglobin gene (HbS) and one gene for thalassemia, a blood disorder that affects hemoglobin production. Individuals with sickle thalassemia can experience a range of symptoms depending on the type and severity of thalassemia. Additionally, compound heterozygous states such as HbSC (one sickle hemoglobin gene and one hemoglobin C gene) or HbSβ+ thalassemia (sickle hemoglobin and beta-thalassemia) also represent distinct genetic forms of sickle cell disease, each with varying clinical manifestations and complications.

In Africa, SCD prevalence is exceptionally high, with an estimated 240,000 affected infants born annually [3]. The SCT is believed to offer a survival advantage against malaria, a factor that has influenced its distribution across malaria-endemic regions of Africa [6,7]. However, this same advantage does not extend to individuals with homozygous sickle hemoglobin (HbSS), who are more susceptible to malaria and its complications.

As a group of autosomal recessive haemoglobinopathies, SCD is characterized by the production of abnormal hemoglobin, which causes red blood cells to become deformed and prone to rupture [8]. This leads to a variety of complications, including severe anemia, organ damage, and chronic pain [9,10]. While there are over 1200 known hemoglobin variants, the most common are HbS, HbC, and HbE, alongside nearly 500 thalassemia mutations that affect the synthesis of globin chains during both prenatal and postnatal development [11]. The multisystemic complications of SCD contribute to its high morbidity and mortality rates, particularly among those with sickle cell anemia (SCA) [12].

The pathophysiology of sickle cell disease (SCD) involves multiple interconnected mechanisms. While the premature destruction of red blood cells—hemolysis—is a hallmark of the disease, the primary cause of morbidity and mortality is vaso-occlusion. This occurs when sickled red blood cells become rigid and adhere to the endothelial lining of blood vessels, obstructing blood flow. These blockages deprive organs of oxygen, leading to tissue ischemia and initiating a cascade of inflammation. This process is further exacerbated by reperfusion injury when blood flow is restored, contributing to additional vascular damage. Additionally, free hemoglobin released during hemolysis binds to nitric oxide, reducing its bioavailability and further impairing vasodilation, which exacerbates vascular occlusion and promotes inflammation [12,13]. These blockages deprive organs of oxygen, increasing the risk of infections, chronic pain, and organ damage, including damage to the kidneys, liver, and spleen. Additionally, hyper-hemolytic crises, common in tropical Africa, are exacerbated by recurrent malaria infections, further complicating the disease’s management in this region [13].

SCD heavily burdens affected individuals and their families, encompassing educational, psychological, financial, and sociocultural challenges. In recognition of this, the United Nations (UN) General Assembly passed a resolution in 2008 acknowledging SCA as a significant public health issue and designated June 19th as World Sickle Cell Day to raise awareness about the disease [14]. The severity of SCD can vary significantly, influenced by factors such as the level of fetal hemoglobin (HbF) and the presence of alpha-thalassemia [15]. HbF, the form of hemoglobin present in fetuses, has a higher affinity for oxygen and does not promote sickling of red blood cells as adult hemoglobin (HbS) does. Higher levels of HbF in individuals with SCD can reduce the frequency of vaso-occlusive episodes and other severe complications. The alpha-thalassemia trait, which results in the reduced production of one or both alpha-globin chains, can also mitigate SCD severity. The presence of alpha-thalassemia reduces the concentration of sickle hemoglobin (HbS) in red blood cells, leading to less sickling and improved red blood cell survival. These genetic modifiers contribute to a more favorable clinical course for some individuals with SCD. While phenotypic variability and environmental and socioeconomic factors are known to play a role, their specific impact on disease severity requires further investigation. Individuals with sickle cell disease often experience diminished health-related quality of life, which can also affect their families.

The treatment of SCA in Africa faces numerous challenges, including limited healthcare infrastructure, inadequate access to essential medications like hydroxyurea, and a shortage of specialized healthcare providers. While hydroxyurea has shown promise in reducing disease severity, its availability and affordability remain significant barriers [16]. Blood transfusions, although crucial for managing acute complications, are often hindered by inadequate blood supply and the risk of transfusion-transmissible infections [17]. Bone marrow transplants, while offering a potential cure, are largely inaccessible due to high costs and procedural complexities. Pain management is frequently suboptimal due to limited access to opioids and associated stigma. Beyond medical interventions, comprehensive care models emphasizing patient education, genetic counseling, and support networks are gaining traction [18]. National sickle cell programs with newborn screening are emerging but remain limited in scope [19]. Despite progress in some areas, the treatment of SCA in Africa continues to be constrained by systemic challenges.

Despite advancements in understanding and treatment, SCA remains a major public health challenge in Africa, necessitating a comprehensive review of its burden, risk factors, and evolving management strategies. Several recent publications, including the Lancet Haematology Commission (2023) [20] and the WHO 2024 Package of Interventions for Sickle Cell Disease Management [21], have provided valuable insights into global strategies for improving SCD outcomes, focusing on key aspects like healthcare delivery, access to medications, and public health interventions. However, while these reports highlight critical recommendations for improving care, there remains a need for a detailed exploration of how these strategies are being implemented and tailored in the African context. This review builds on these foundational works by providing a region-specific, comprehensive synthesis of SCD in Africa, with a particular focus on clinical outcomes, management practices, and healthcare system implications within African nations.

This review aims to provide a comprehensive overview of sickle cell anemia in Africa, addressing the following key research questions: (i) What is the burden of SCA in Africa? (ii) What are the reported clinical presentations observed in African populations? (iii) What documented lessons and best practices exist for managing SCA in Africa? Understanding SCA’s prevalence, mortality rates, and socioeconomic effects is crucial for informing effective interventions. Identifying risk factors and clinical manifestations can aid in early diagnosis and targeted prevention strategies. Examining successful treatment approaches, challenges, and emerging therapies can inform evidence-based practices. This review aims to contribute to a deeper understanding of SCA in Africa and provide valuable insights for researchers, healthcare professionals, and policymakers working to improve the lives of individuals affected by this disease.

## 2. Methods

### 2.1. Study Design

The methodology adopted for this critical literature review was informed by the guidelines outlined by Jesson and Laccy [22] for conducting such reviews. This study aimed to extend beyond a simple overview of existing research and contribute conceptually and theoretically to understanding the burden of SCA in Africa, identify specific risk factors and clinical presentations in African populations, and document lessons and best practices for treatment in the region.

### 2.2. Search Strategy

This review employed a systematic approach to identify and analyze relevant literature on SCA in Africa. A comprehensive search was conducted using the PubMed and Google Scholar databases, focusing on articles published between 2018 and 2024. Additionally, we searched the WHO website and the Sickle in Africa site (https://www.sickleinafrica.org/distinct_publications_group (accessed on 30 October 2024)). Key search terms included “sickle cell disease”, “burden”, “interventions”, “challenges”, and “sub-Saharan Africa”.

### 2.3. Inclusion Criteria

The inclusion criteria for selected articles were as follows:

Research Question 1:
Focus: Studies specifically addressing SCA in sub-Saharan Africa.
Research Type: Original research articles, including quantitative and qualitative studies.
Publication Language: English.
Research Questions 2 and 3:
Focus: Studies specifically addressing SCA in sub-Saharan Africa.
Research Type: Original research articles, including quantitative and qualitative studies, review articles, reports, expert opinions, and commentaries.
Publication Language: English.

### 2.4. Screening, Selection, Data Abstraction, and Analysis

A comprehensive literature search was conducted between 1 and 13 September 2024, to identify relevant studies. Three independent researchers (HWM, PGI, and ES) collaborated using reference management software, Covidence (Covidence systematic review software, Veritas Health Innovation, Melbourne, Australia; available at www.covidence.org) to ensure a streamlined and unbiased process. This software facilitated duplicate removal across databases, resulting in a clean reference list. Additionally, it enabled blinded voting during the article screening and selection stages, enhancing objectivity and efficiency. Data extraction from the selected articles was performed using a standardized form, capturing information on authors, publication year, study design, geographical region, sample size, population characteristics, and key findings related to SCA burden, risk factors, and interventions.

Following the initial search and screening, qualitative data analysis software NVivo (QSR International, NVivo 14 for Windows., QSR International Pty Ltd, Burlington, MA, USA, 2023) was employed to analyze the retrieved full-text articles. This software was employed to organize research findings, explore emerging themes, and extract key insights relevant to the research questions. Within the software, articles were imported and categorized based on pre-defined codes aligned with each research question (e.g., “Burden of Sickle Cell Disease in Africa”, “Risk Factors and Clinical Presentations”, “Lessons Learnt”). These codes were further subdivided into sub-codes for capturing specific themes, such as “ Early Diagnosis and Screening”, “Comprehensive and Holistic Care”, or “Barriers to Access and Affordability”. Textual data relevant to these themes were coded, enabling efficient retrieval and analysis across different studies. The software’s query function facilitated the identification of patterns and connections between themes. Additionally, visualization tools generated mind maps and concept maps, allowing for a visual representation of thematic relationships and fostering critical interpretation. Reflections and critical analyses were documented within the software using the memo functionality. These notes guided the synthesis of results and the development of a comprehensive and insightful final review. Key findings were synthesized and presented in a narrative format, supported by relevant figures and tables.

## 3. Results

This initial search yielded 737 articles published between 2018 and 2024. After removing duplicates and applying rigorous screening criteria, 36 articles were selected for in-depth review.

Research Question 1: Burden of sickle cell anemia in Africa

Nineteen primary studies [23,24,25,26,27,28,29,30,31,32,33,34,35,36,37,38,39,40,41] were included in this research question. Of these, three were conducted in Southern [30,38,40], five in Western [23,26,28,41], and twelve in Eastern Africa [24,25,27,29,31,32,33,34,35,36,37,39]. More details are presented in Table 1.

The burden of SCD in Africa is substantial, with variations in prevalence across different countries and age groups. Newborn screening studies, such as those from Mali [23], DRC [36], and Angola [30], reveal a high prevalence of SCD at birth, highlighting the need for early diagnosis and management. For children, Uganda [24] and Tanzania [32,33] provide evidence of significant prevalence, emphasizing the importance of targeted interventions for this age group. Data from Malawi [40] and Nigeria [28] suggest that SCD continues to impact older children, further underlining the need for ongoing care and support.

### 3.1. Newborns

In studies focusing on newborns, the prevalence of SCD is notably high across several African countries. For instance, Guindo (2024) reported a prevalence of 1.64% among 3676 newborns in Mali [23], highlighting the significant number of newborns affected by SCD at birth. In the Democratic Republic of the Congo (DRC), Katamea (2023) found that 5.01% of newborns had hemoglobin SS (HbSS), which indicates SCD [36], while Kasai (2023) observed a prevalence of 2.2% for HbSS among 1432 newborns in the DRC [37]. Additionally, Olaniyan (2023) reported that 15.7% of newborns in Angola had results consistent with SCD [30].

### 3.2. Children and Young Adults

When examining the prevalence of SCD among children, regional variations are evident. In Uganda, Datta (2023) found a prevalence of 3.2% among 718 children aged 18 months to 12 years [24], while Bello-Manga (2020) reported a prevalence of 3.9% of severe anemia in children aged 5 to 12 years with SCD in Nigeria [28]. In Tanzania, Ally (2023) observed a prevalence of 28.3% among children aged 0–5 years [32] while Malawi also reports significant figures, with Chimbatata (2021) noting that SCD represented 3.1% of the total patients aged seven weeks to twelve years [40]. Data on older age groups, though less frequent, are still important. In Uganda, Namukasa (2024) found that 5.8% of students aged 17–20 years had sickle cell trait [39].

Research Question 2: Risk factors and clinical presentations observed in African populations

Ten publications were included under this research question [40,42,43,44,45,46,47,48,49,50]. Nine were journal articles [40,42,44,45,46,47,48,49,50], and one was a book chapter [43]. This review revealed that the clinical presentations of SCA in African populations are diverse. We grouped them into three primary themes: acute complications, chronic complications, and biological and demographic associations. Table 2 presents more details.

Theme 1: Acute Complications

Acute manifestations of SCA are critical as they often lead to emergency care and immediate risk of mortality. Key acute symptoms include vaso-occlusive crisis, acute chest syndrome, stroke, and neurological events.

- Vaso-occlusive Crisis (Painful Crisis): This is the most common acute manifestation across studies. Isa (2020) found that 60% of patients experienced bone pain crises [44], while Hassan (2022) noted that 43.4% of admitted patients presented with bone pain [50]. Painful crises can affect various parts of the body, including bones, joints, and the abdomen, as highlighted by Adegoke (2020) [43].
- Acute Chest Syndrome: A life-threatening condition identified in multiple studies, with Isa (2020) reporting a 5.9% occurrence rate and Mbayabo (2023) confirming its role as a severe pulmonary complication [46].
- Stroke and Neurological Events: Stroke was a significant concern, reported by Isa (2020) and Mbayabo (2023), with incidence rates varying across populations [44,46]. Isa (2020) reported a 2.5% incidence of stroke, while Mbayabo (2023) emphasized its prevalence (2.94%), especially among males.
- Other Acute Presentations: Other notable acute symptoms included anemia-related complications such as jaundice, tachypnea, and altered mental status [42,49], with some cases of splenic sequestration and priapism, particularly among males [47,48].

Theme 2: Chronic Complications

SCA is characterized by several long-term complications that significantly impact patient quality of life. This review revealed leg ulcers and avascular necrosis, kidney and liver disease, pulmonary hypertension, and other organ damage as common clinical presentations of the disease.

- Kidney and Liver Disease: Renal complications were highlighted by both Adebayo (2022), who focused on chronic kidney damage, and Duru (2021), where nephropathy was reported [45,48]. Hepatomegaly and splenomegaly were also observed as chronic complications in Sahli’s (2023) cohort in Tunisia [49].
- Pulmonary Hypertension and Other Organ Damage: Isa (2020) and Duru (2021) noted pulmonary hypertension in their patient populations [44,48]. Chronic vasculopathy leading to tissue damage and organ dysfunction was reported by Adebayo (2022) [45].

Theme 3: Biological and Demographic Associations

This review revealed that biological markers, age, and sex influenced SCA severity and symptom presentation.

- Hemoglobin Levels and Disease Severity: Low fetal hemoglobin (HbF) levels were correlated with increased disease severity [46,47]. Studies also reported that disease severity worsened with age, particularly during school years [46], while males generally had more severe disease [47].
- Anemia and Reticulocyte Count: Normocytic normochromic anemia was a common finding, with patients having a high reticulocyte count, indicating compensatory erythropoiesis [46,49].
- Gender Differences: Males experienced more severe disease in several areas, including the frequency of leg ulcers and priapism, as reported by Lumbala (2022) [47] and Duru (2021) [48], while females were associated with higher HbF levels, which were inversely correlated with disease severity [46].

In summary, the clinical presentation of sickle cell anemia in African populations is marked by a spectrum of acute and chronic complications, with painful crises, leg ulcers, and organ damage being prevalent. Biological factors like HbF levels, as well as demographic factors such as age and sex, influence disease severity and outcomes, underscoring the need for tailored clinical interventions.

Research Question 3: Lessons and best practices for management of SCA in Africa

Eight articles were included under this research question [51,52,53,54,55,56,57,58]. Of these, four were commentaries [51,54,55,58], and four were reviews [52,53,56,57]. Three themes emerged for best practices and three for lessons learned in the management of SCA in Africa. Table 3 presents more details.

### 3.3. Best Practices

Theme 1. Early Diagnosis and Screening

- Newborn Screening (NBS): Across many sources [53,55,56], the emphasis on early diagnosis via NBS emerges as a cornerstone of managing SCD in Africa. Early identification enables timely interventions, which can significantly reduce mortality. Countries such as Ghana, Nigeria, and Uganda have successfully integrated NBS into existing healthcare frameworks, though coverage is still limited across the continent.
- Point-of-Care Testing (POCTs): The use of simple, cost-effective, and accurate tests like SickleScan™ and HemoTypeSC™ has proven particularly beneficial in low-resource settings [53,58]. These rapid tests allow for large-scale implementation and are key to the success of early diagnosis programs.
- Genetic Counseling: Akisente (2022) [55] underscores the importance of accessible genetic counseling for at-risk couples. This ensures families understand inheritance patterns and potential risks, which supports informed decision-making and early family engagement in disease management.

Theme 2. Comprehensive and Holistic Care

- Multidisciplinary Approach: Many authors [51,56,57] stress the importance of a holistic approach involving medical and psychosocial interventions. The effective management of SCD requires addressing not only the physical symptoms but also mental health and social aspects, which include patient education, lifestyle changes, and emotional support through community groups.
- Pharmacotherapy: Hydroxyurea is highlighted as a cost-effective and widely used disease-modifying therapy across Africa [52,56,57]. It has been effective in reducing complications like vaso-occlusive crises and strokes. Newer therapies (e.g., crizanlizumab, L-glutamine, voxelotor) are emerging but remain costly and less accessible [53].
- Supportive Care and Education: SCD clinics in Tanzania and Nigeria offer comprehensive services such as pain management, blood transfusions, and infection prevention [56]. Patient education and caregiver support play pivotal roles in ensuring adherence to treatment plans and lifestyle modifications [52,57].

Theme 3. Collaboration and Resource Utilization

- International Networks: Collaborative projects like the Consortium on Newborn Screening in Africa (CONSA) and partnerships between healthcare providers, policymakers, and community organizations have been crucial in addressing SCD challenges [53,56]. Global partnerships provide technical support and financial assistance, ensuring the sustainability of key programs.
- Local Solutions: Olukorede (2022) discussed leveraging locally available resources, such as medicinal plants with anti-sickling properties [54]. This approach is essential for making treatments more accessible in low-resource settings. Innovative research conducted in Africa should focus on affordability and be sensitive to local cultural contexts.
- Training Healthcare Workers: Continuous training on SCD management, including emerging therapies like hydroxyurea, is essential for improving care outcomes [55]. Training ensures that healthcare workers are equipped with up-to-date knowledge of diagnosis, treatment, and patient care, especially in rural or under-resourced areas.

### 3.4. Lessons Learned

Theme 1. Barriers to Access and Affordability

- Cost of Treatment: One of the most significant lessons from across studies is the prohibitive cost of advanced therapies, including gene therapy and hematopoietic stem cell transplantation (HSCT), as noted by Esoh (2021) [51] and Coetzee (2022) [52]. While these treatments show promise, their affordability remains a significant concern, and many patients cannot access them.
- Limited Resources: Many African countries struggle with resource constraints that limit the availability of diagnostic tools, medications, and trained healthcare professionals [55,56]. This under-resourced healthcare infrastructure complicates the rollout of best practices such as NBS and comprehensive care.
- Data Scarcity: A lack of accurate data on SCD prevalence and outcomes continues to undermine advocacy and policy development [54,56]. Without robust data, it is challenging to secure funding, attract international research collaboration, or develop tailored treatment protocols for the African context.

Theme 2. Cultural and Community Challenges

- Stigma and Misconceptions: There is widespread stigma around SCD in many communities, often fueled by myths and misconceptions about the disease being a divine punishment or witchcraft [53]. These cultural barriers impede early diagnosis and adherence to treatment plans. Public sensitization efforts led by healthcare professionals and NGOs are critical for combating these misconceptions.
- Community Involvement: Engaging families and communities is essential for improving outcomes [58]. Education campaigns, particularly those led by local NGOs like the Sickle Cell Foundation Nigeria, have effectively raised awareness about SCD and encouraged participation in screening and treatment programs [56].
- Patient Participation in Research: Low participation in clinical trials remains a significant challenge [54]. Research in Africa often faces hurdles related to patient enrolment, which are further compounded by poor communication with potential participants and a lack of culturally appropriate engagement strategies.

Theme 3. Innovative Solutions for Sustainability

- NBS Sustainability: Oron (2020) and Egesa (2022) emphasize that newborn screening programs, though proven effective, need long-term financial backing and more robust government commitment to remain sustainable [53,58]. Pilot programs have shown that integrating NBS into national healthcare frameworks can be successful, but expansion across the continent requires more sustained political will.
- Gene Therapy and Emerging Treatments: Coetzee (2022) notes that ongoing research into gene therapy and other advanced treatments is essential for the future of SCD management [52]. However, such innovations should be accompanied by strategies to reduce costs and improve access. Without addressing these affordability issues, these advancements will remain out of reach for most African populations.
- Improving Care Models: Lessons from successful programs in Tanzania and Nigeria suggest that integrating SCD care with national health insurance schemes or partnering with global organizations can significantly increase access to affordable care, even in rural areas. Sustainability will depend on broader health system reforms and investment in health infrastructure [56].

## 4. Discussion

Sickle cell anemia in sub-Saharan Africa continues to carry the majority of the global burden [59], creating a host of acute and chronic clinical complications for patients. SCD also has a geographical burden, as it appears to be more concentrated in the Eastern and Western parts of Africa, which is conducive to the malaria endemic in those areas. The burden of SCA in Africa is significant, with varying prevalence across countries and age groups. Clinical presentations in African populations are diverse, ranging from acute complications like vaso-occlusive crises and stroke to chronic conditions such as avascular necrosis and pulmonary hypertension. These findings correlate with the conclusions made by the Global Burden of Disease Study carried out in 2021 [60], where global total births of children with SCD increased, driven by increased numbers in the Caribbean and western, central, and sub-Saharan Africa. This indicates that Africa is indeed a priority population for focused interventions against global SCD morbidities. Chronic complications observed in several studies are most likely due to constant sickling of the HbS erythrocytes, which progressively injure and cause tissue damage in vital organs like the liver, spleen, and kidneys [61].

Disease severity is influenced by biological markers like HbF, age, and sex, with males experiencing more severe disease manifestations, including leg ulcers and priapism. HbF inhibits the polymerization of HbS. Sickle red blood cells with elevated levels of HbF exhibit a survival advantage, lasting five to seven times longer than those with lower HbF concentrations, which correlates with reduced morbidity and mortality in children [62]. Increasing age is positively associated with the severity of SCD because of complications that develop from the disease [63]. One proposed explanation for the heightened disease severity in males is the elevated estrogen levels in females, which promote nitric oxide production and inhibit its degradation. Nitric oxide’s association with the transcriptional regulation of HbF may play a role in the observed gender differences in HbF expression, which is usually elevated in females [64].

Effective management strategies emphasize early diagnosis through NBS, accessible genetic counseling, and a holistic approach that addresses both medical and psychosocial needs. NBS has seen positive outcomes in North America [65], where it is integrated as part of routine universal public health programs. This early intervention allows for timely medical management, including the administration of penicillin prophylaxis, vaccinations, and early transfusion therapy, which has been shown to significantly reduce mortality and improve quality of life for affected children. Similar initiatives have been implemented in Africa with less effective results due to limited coverage [53,55,56]. Best practices in countries like Ghana and Uganda integrate NBS into existing healthcare frameworks, and the use of rapid point-of-care tests has proven effective in low-resource settings. A 2022 review suggested that the combination of both birth screening and immunization centers could be a game-changer [66]. The high prevalence of SCD strains the healthcare facilities in the regions. Although NBS is available in a few countries, it requires highly qualified medical professionals who can interpret the blood morphologies of patients. For these samples to be analyzed, they require the appropriate equipment.

In addition to newborn screening, early treatment strategies, including the use of hydroxyurea, blood transfusions, and even stem cell transplantation, are essential components of effective management. These treatments can dramatically reduce complications and improve survival rates. However, access to these therapies remains limited in sub-Saharan Africa, primarily due to financial constraints, shortages of trained healthcare professionals, and inadequate healthcare infrastructure. The emergence of gene therapies, while offering the potential for curative treatment, is currently out of reach for most patients in low-resource settings, given the high costs and complex medical requirements. Nonetheless, combining NBS with early intervention programs remains the most viable strategy for improving outcomes for children with SCD in these regions.

Lessons from successful programs highlight the potential for international partnerships to increase access to care and improve outcomes for individuals living with SCD in Africa. Global partnerships [67], led by organizations like the Sickle Cell Disease International Organization (SCDIO) and the Global Sickle Cell Disease Network (GSCDN), have significantly advanced SCD management through educational programs, training workshops, and community outreach. These collaborations have improved access to quality care, enhanced clinical practices, and increased public awareness in regions heavily impacted by SCD.

Despite these efforts, significant challenges persist, including high costs of advanced therapies, resource constraints, and cultural stigma. Gene therapy has emerged as a promising treatment for SCD, offering the potential for a curative approach by modifying the patient’s own hematopoietic stem cells to produce normal hemoglobin. Several gene therapy modalities, such as gene editing (e.g., CRISPR-Cas9) and gene addition (e.g., lentiviral vector-based therapies), have shown significant success in clinical trials, leading to sustained reductions in disease severity and even remission in some patients. However, while these therapies hold great promise, their high cost, complex infrastructure requirements, and specialized medical expertise required leave them currently inaccessible in many African settings. In addition, the lack of reliable healthcare systems, as well as limited access to advanced medical technologies and post-treatment care, further restricts their applicability in sub-Saharan Africa. As such, while gene therapy represents a major step forward in the treatment of SCD, it remains an option primarily for patients in high-resource countries, with its potential in Africa still being hindered by significant barriers.

Sustainable care models require strong government commitment; thus, we recommend that local health authorities work closely with governments to increase political will and assist with allocating resources for SCD and related challenges. It is crucial to integrate clinical management of SCD with mental health, social and cultural aspects, awareness campaigns, and emotional support through community groups in areas with a high incidence of SCD.

In Africa, where resources are generally limited, integration of SCD initiatives into national health systems can be a useful mechanism to amplify the reach and impact of interventions while leveraging existing systems and infrastructure. We recommend prioritizing this approach to enhance the efficiency and sustainability of SCD care. Further investment in healthcare infrastructure is crucial to strengthen service delivery across all levels, while research into locally available resources, such as medicinal plants with anti-sickling properties, should be expanded to develop cost-effective and culturally relevant treatment options.

One limitation of this review is that it exclusively includes studies published in English. As a result, important research from non-English-speaking regions, particularly Francophone and Lusophone countries, may not have been fully captured. Notably, there is a significant body of work on sickle cell disease in Francophone countries such as Mali and the Democratic Republic of Congo (DRC), as well as in Portuguese-speaking Angola, which could provide valuable insights into regional differences in sickle cell care. Studies such as the CADRE study and research conducted by INSERM and the Fondation Pierre Fabre have contributed significantly to the understanding of sickle cell disease in these regions. The absence of these studies may limit the comprehensiveness of our review in reflecting the full spectrum of sickle cell care across sub-Saharan Africa. Future reviews should consider including research in other languages to provide a more complete and diverse understanding of sickle cell care and its regional variations. Another limitation of the review is the potential sample bias introduced by the inclusion of studies from referral centers, where the incidence of severe complications may be higher compared to population-based studies. This could lead to an overrepresentation of more severe cases in our findings. The findings should, therefore, be interpreted with this limitation in mind. The differences in study design and setting may introduce some uncertainty in the generalizability of the findings to broader populations, particularly in less specialized healthcare settings. Future studies could benefit from incorporating data from both referral centers and population-based studies to provide a more balanced perspective on the incidence of severe complications in sickle cell disease.

## 5. Conclusions

The high burden of SCD in Africa underscores the urgent need for targeted, comprehensive interventions to address the diverse clinical presentations and complications associated with the disease. Although there have been promising initiatives, such as newborn screening and international partnerships, their effectiveness remains limited by resource constraints, cultural barriers, and a lack of integration into national health systems. Sustainable solutions require a multi-faceted approach that includes strengthening healthcare infrastructure, increasing political commitment, and fostering community-based support. African countries can enhance the reach and impact of SCD interventions through leveraging existing healthcare frameworks and exploring culturally relevant treatments. Ultimately, a combination of innovative care models, investment in research, and a collaborative effort among stakeholders is critical to improving the quality of life and survival outcomes for individuals living with SCD across the continent.

## Figures and Tables

**Table 1:** Burden of sickle cell disease.

Lead Author, Year	Country	Region	Sample Size	Age Group	Prevalence	Mortality Rate	Other Relevant Data
Guindo, 2024 [23]	Mali	Western	3676 newborns	newborn	Among 3676 births, 1.64% had SCD; 21.79% were SCD carriers	Estimated 50–80% before 5 years of age without care management	Adaptation of Routine SCD NBS was acceptable
Datta, 2023 [24]	Uganda	Eastern	718	18 months to 12 years	3.20%	Cerebral malaria, 16 died (8.8% mortality)	Given the resource demands of preventive approaches, using objective CNS biomarkers, assessed with a semi-automated bench-top device is a logical step for managing children with severe malaria
Adan, 2023 [25]	Uganda	Eastern	600 children with severe malaria and 185 children with sickle cell disease	Not covered		Among children with SM, the prevalence of AKI on admission was 44.2%, and 7.3% of children died and Among children with SCD, the prevalence of AKI on admission was 23.2%, and 3.2% of children died	Children with SM had more severe AKI and signs of disease severity with a higher frequency of coma and respiratory distress, while children with SCD were more likely to have severe anemia and hyperfiltration
Ladu, 2023 [26]	Nigeria	Western	182 SCD patients and 102 healthy controls	SCD 1–45 years and Control 1–32 years	182 SCD patients (175 Hb SS, 5 Hb SC, and 2 Hb SB thal) and 102 controls (93 Hb AA, 7 Hb AS, and 2 Hb AC)	Not covered	Utility of light microscopy in the assessment of red cells containing HJB and AI inclusions as indices of splenic dysfunction in Nigerian SCD patients
Mikobi, 2023 [27]	DRC	Eastern	Follow-up of 1112 sickle cell pregnant women, with 146 who met inclusion criteria; 120 in homozygous AA pregnant control group	17 to 34 years	HbS level was 90.2%, HbF 7.6%, and HbA2 2.2%		Study revealed that the hemodynamic changes during pregnancy, essentially the progressive decrease in hematocrit and the increase in plasma volume, are responsible for anemia by hemodilution, and these changes are responsible for hypoxia during pregnancy
Bello-Manga, 2020 [28]	Nigeria	Western	Study included 941 children with sickle cell anemia; defined as phenotype HbSS or HbSβ0 thalassemia	5 years–12 years	3.9% of severe anemia	Not covered	Severe anemia is common in children with SCA greater than five years of age living in northern Nigeria
Namugerwa, 2023 [29]	Uganda	Eastern	372	31–40 years	44.09% had good knowledge	Not covered	Study revealed that there is a high level of general awareness about SCD/SCA but comprehensive knowledge about its cause and prevention was low and the majority did not find a reason as to why it should influence their marital decisions
Olaniyan, 2023 [30]	Angola	Southern	2000 babies	≤6 months	Out of 1000 mothers, 70 (3.5%) reported a family history of SCD. Among them, 11 (15.7%) had results consistent with SCD (FS), and 28 (40%) had results consistent with the sickle cell trait (FAS).		Study demonstrates the real-world feasibility and accuracy of POC tests to screen infants for SCD in Angola. This study also suggests that including vaccination centers may improve the capture rate for early infant SCD screening programs. In terms of feasibility and how easy to use, POC—nurses (48.5%), students (30.5%), laboratory technicians (20.8%), and physicians (0.2%)
Aimé, 2024 [31]	DRC	Eastern	448	Median age was 48 months with the extreme ages 2 and 59 months	12.7% sickle cell disease	Not covered	Study highlights that the burden of sickle cell disease in pediatric hospitals, particularly among children under 5, is underestimated due to the lack of systematic screening
Ally, 2023 [32]	Tanzania	Eastern	364	0–5 years	28.30%	Not covered	Among the positively tested with SCD, the majority were female at 71.7% (65/91), whereas males were 28.3% (26/91)
Ambrose, 2020 [33]	Tanzania	Eastern	17,200	0–24 months	Prevalence of sickle cell trait and disease of 20.3% (3492/17,200) and 1.2% (210/17,200), respectively	Not covered	Our district-level data will guide public health policy by targeting screening and hydroxyurea therapy to areas with a high prevalence, until universal newborn screening becomes available
Khisa, 2021 [34]	Kenya	Eastern	350 blood donors and 10 patients transfused with sickle cell trait	Over 16 years	Prevalence of the sickle cell trait: 14.28%	Not covered	No clinical abnormalities in patients transfused with sickle cell trait; however, it was observed that acquired hemoglobin AS (sickle cell trait) was detected among the transfused population
Adam, 2019 [35]	Sudan	Eastern	400	0–18 years	14.8% Sickle cell trait patients were 11.3% Sickle cell disease-positive patients were 3.5%	Not covered	NA
Katamea, 2023 [36]	DRC	Eastern	588 newborns screened	newborn	Among the newborns, 68.59% had hemoglobin AA (HbAA), 26.21% had hemoglobin AS (HbAS), 5.01% had hemoglobin SS (HbSS), and 0.19% had hemoglobin AC (HbAC)	Not covered	NA
Kasai, 2023 [37]	DRC	Eastern	1432	1432 babies	The incidence at birth was 2.2% for HbSS homozygosity and 21% for HbAS heterozygosity	Not covered	Setting up a neonatal screening program and an awareness unit is necessary to assess the need for care services correctly
Mano, 2024 [38]	Namibia	Southern	202	Baby born	9.40%	Not covered	Future studies should therefore expand the facilities, consider community-based NBS, and include all newborn babies regardless of gestational age to obtain an accurate representative birth prevalence of SCD and SCT in Namibia
Namukasa, 2024 [39]	Uganda	Eastern	399 students	17–20 years	5.8% of participants who were tested during this study had SCT	Not covered	Only 29 (7.3%) participants knew of a family member with sickle cell
Chimbatata, 2021 [40]	Malawi	Southern	512	Aged 7 weeks to 12 years	Out of 512 patients with sickle cell disease (SCD), representing 3.1% of the total, 13.3% were newly diagnosed cases. Only 13.2% of these were diagnosed during infancy	Not covered	NA
Oppong, 2020 [41]	Ghana	Western	938	1–12 years	prevalence of sickle cell disorders at 2.0%	Not covered	NA

**Table 2:** Clinical presentation.

Author, Year	Country	Region	Sample Size	Age Group(s)	Clinical Presentations	Other Relevant Data
Sawe, 2019 [42]	Tanzania	Eastern	752	6–23 years	Tachypnea, fever, hypoxia, altered mental status, and bradycardia	The overall morality (emergency department plus inpatient) was 2.1%. Overall, 50% of deaths’ occurred within 24 h of emergency department presentation
Adegoke, 2020 [43]	N/A	N/A	N/A	Children	Painful Crises: These are the most common symptom and involve severe pain in the bones, joints, muscles, and abdomen. They occur when sickle cells block blood flow to these areas. Anemia: The sickle-shaped cells are destroyed more rapidly than normal red blood cells, leading to anemia. This can cause fatigue, shortness of breath, and pale skin. Splenic Sequestration: The spleen can become enlarged and trap sickle cells, leading to abdominal pain and a sudden drop in red blood cell count. Stroke: Sickle cells can block blood flow to the brain, leading to stroke. Acute Chest Syndrome: This is a life-threatening complication that can cause chest pain, fever, and difficulty breathing. Organ Damage: Over time, sickle cell anemia can damage various organs, including the heart, liver, kidneys, and lungs	None
Isa, 2020 [44]	Nigeria	Western	3622	≤15	60%, 23.8%, 5.9%, 4.8% and 2.5% had a bone pain crisis, dactylitis, acute chest syndrome, priapism, and stroke, respectively. The most frequent chronic complications were leg ulcers (6.5%), avascular necrosis of bone (6.0%), renal (6.3%) and pulmonary hypertension (1.1%)	Only 13.2% had been hospitalized while 67.5% had received blood transfusion
Adebayo, 2022 [45]	DRC	Central	361	2–18 yrs	Recurrent episodes of ischemia–reperfusion injury and chronic vasculopathy, which lead to acute and/or chronic tissue damage and organ injuries or dysfunction, including kidney disease	None
Mbayabo, 2023 [46]	DRC	Central	136	6 months–18 yrs	Key Clinical Features: Normocytic normochromic anemia: A reduction in red blood cells with normal size and hemoglobin content. High reticulocyte count: Increased production of new red blood cells to compensate for the anemia. Signs of hemolysis: Breakdown of red blood cells, leading to jaundice and other related symptoms. Chronic complications: These can include the following: Stroke: Particularly in males. Avascular bone necrosis: Damage to bone tissue due to lack of blood flow. Leg ulcers: Open sores on the legs. Acute chest syndrome: A serious lung condition. Disease Severity: Disease severity tends to be higher in males: However, this difference was not statistically significant. Disease severity increases with age: Children of school age (6–10 years) had significantly higher severity scores than adolescents (10–18 years). Biological factors associated with disease severity: Neutrophil count: Higher in moderate versus severe disease. Fetal hemoglobin (HbF) levels: Inversely correlated with disease severity.	Higher levels of direct bilirubin and creatinine in boys. Higher HbF levels in girls. Inverse correlation between HbF levels and disease severity. Decrease in HbF levels with age.
Lumbala, 2022 [47]	DRC	Central	166	18–40 yrs	Disease Severity: Majority of patients (64.5%) had moderate severity. Severity increased with age (*p* ≤ 0.001). Males tended to have more severe disease compared to females (*p* = 0.012). Low HbF levels were associated with increased severity (*p* = 0.015). Malnutrition: Present in 47% of patients. More frequent in males (aOR 3; *p* = 0.006). Associated with severe phenotype (aOR 7.21; *p* = 0.001). Chronic Complications (33.7% of patients): Leg ulcers (10.8%)—More frequent in males (*p* = 0.001). Hip disease (15%)—More frequent with increasing age (*p* = 0.023). Neurological events (2.4%). Priapism (8% of males)—Associated with increased severity (*p* = 0.045). Biological Features: Normocytic normochromic and regenerative anemia. Low fetal hemoglobin rate (7.3 ± 5.5%). No correlation between HbF and age (*p* = 0.845)	Sex differences: Higher HbF levels in females. More severe disease in males.
Duru, 2021 [48]	Nigeria	Western	270	16–55 yrs	68 had leg ulcers, 43 of the males had priapism (erectile dysfunction in 8), 42 had AVN, 31 had nephropathy, 23 had osteomyelitis, 15 had osteoarthritis, 12 had cholelithiasis, 10 had stroke or other neurological impairment, 5 had pulmonary hypertension, while 23 had other complications. Frequency of crisis ranged from 0 to >10/year, with a median of 2. Of the 219 recorded, 148 of the patients had been transfused in the past, while 71 had not	None
Sahli, 2022 [49]	Tunisia	North	66	1–49 yrs	The study found that sickle cell anemia (S/S) patients had lower baseline hemoglobin levels and were more likely to experience jaundice, mucosal skin pallor, hepatomegaly, and splenomegaly compared to other sickle cell disease genotypes (S/C, S/β-thalassemia, S/OArab). Vaso-occlusive attacks and worsening anemia were the most common acute complications, while cholelithiasis was the most common chronic complication. Overall, S/C patients had the best clinical outcomes and were less affected by chronic complications, suggesting that they may lead a more normal life compared to other genotypes	None
Hassan, 2022 [50]	Nigeria	Western	173	3–17 yrs	Of the patients admitted, 43.4% presented with bone pain, 21.4% with jaundice, and 11.6% with abdominal pain. Some patients exhibited multiple symptoms	None
Chimbatata, 2021 [40]	Malawi	Southern	513	Up to 9 yrs	Anemia (94.1%), sepsis (79.5%), and painful crisis (54.3%) were the most recorded clinical features. Leg ulcers, priapism, and dactylitis were the least common clinical features, representing 0.6%, 1.0%, and 0.6%, respectively. In addition, 68 (16.4%) patients had malaria, diagnosed using a rapid diagnostic test	None

**Table 3:** Best practices and lessons learned in the management of sickle cell anemia in Africa.

Author, Year	Article Type	Best Practices	Lessons Learnt
Esoh, 2021 [51]	Commentary	Early Intervention: Neonatal screening and comprehensive care are essential for reducing mortality rates. Holistic Approach: Addressing both medical and psychosocial aspects of sickle cell disease is crucial for effective management. Collaboration: International networks and collaborations are essential for sharing knowledge, resources, and best practices.	Affordable Care: Hydroxyurea and hematopoietic stem cell transplantation, while effective, need to be made more accessible and affordable for African populations. Quality of Life and Increased Life Expectancy: As mortality rates decline, improving quality of life and increasing life expectancy for individuals with sickle cell disease should be prioritized.
Coetzee, 2022 [52]	Review	Multifaceted Approach: Treatment plans should be tailored to individual needs and include a combination of medications, lifestyle modifications, and supportive care. Early Intervention: Prophylactic antibiotics and regular monitoring, especially for children, can help prevent serious complications. Lifestyle Modifications: Staying hydrated, maintaining a healthy diet, avoiding triggers, and managing stress are essential for managing sickle cell disease. Supportive Care: Access to patient support groups and community resources can provide emotional and practical support.	Gene Therapy and Stem Cell Transplantation: These are potential curative therapies, but they come with significant risks and may not be accessible to all. Ongoing Research: Continued research is essential for developing innovative treatments and finding a cure for sickle cell disease. Individualized Care: Treatment plans should be individualized based on the patient’s specific needs and circumstances.
Egesa, 2022 [53]	Review	1. Newborn Screening (NBS) Programs: Early diagnosis through newborn screening is essential to initiate comprehensive care that can be life-saving. However, NBS is not yet universal in sub-Saharan Africa, despite proven efficacy in developed countries. Expanding these programs by integrating them into existing healthcare infrastructure (e.g., immunization programs or HIV-EID initiatives) has proven feasible and sustainable in countries like Ghana, Nigeria, and Uganda. Collaborations like the Consortium on Newborn Screening in Africa (CONSA) aim to address this gap through pilot projects in several countries. 2. Use of Point-of-Care Tests (POCTs): Rapid, cheap, and accurate POCTs such as HemoTypeSC™ and Sickle SCAN™ have proven effective in diagnosing SCD in newborns. These tests are crucial for wide-scale screening as they require minimal training and resources, and they are particularly well-suited for low-resource settings in Africa. 3. Hydroxyurea as Standard Care: Hydroxyurea, a century-old drug, remains the most widely used disease-modifying therapy for SCD in Africa. Though other new therapies have emerged (L-glutamine, crizanlizumab, voxelotor), hydroxyurea’s affordability and availability make it the mainstay treatment in most African countries. 4. Hematopoietic Stem Cell Transplant (HSCT): HSCT is currently the only curative therapy for SCD, though it remains underutilized in Africa due to the cost and complexity. Its use should be scaled where resources permit, especially in partnership with global organizations to subsidize costs.	1. Addressing Myths and Misconceptions: Misconceptions around the causes and management of SCD, such as beliefs in witchcraft or divine punishment, undermine proper health-seeking behavior. Continuous public sensitization, led by clinicians and Ministries of Health, is critical to dispelling these myths and improving the understanding of SCD as a genetic disorder. 2. Comprehensive Family Support: Families need psychosocial support to cope with an SCD diagnosis. This includes face-to-face counseling, ongoing education about the disease, and addressing stigmatization in communities. Emphasis should be placed on engaging parents early, offering genetic counseling, and encouraging active management and care for their children. 3. Challenges with Health Infrastructure: Widespread implementation of effective treatment and screening programs in Africa is hindered by resource constraints, limited laboratory infrastructure, and high costs of standard methods like high-performance liquid chromatography (HPLC). Expanding POCT use and prioritizing NBS in national healthcare strategies are crucial for sustainable long-term care improvements.
Olukorede, 2022 [54]	Commentary	Utilize existing resources: Explore the potential of readily available medicinal plants with proven anti-sickling properties. Prioritize affordability and accessibility: Develop treatments that are financially viable and easily obtainable for patients in resource-limited settings. Focus on patient participation: Conduct clinical trials in Africa with clear communication and respect for cultural practices to increase patient enrollment.	Limited investment: Low financial resources in Africa hinder research and development of new drugs. Complex disease: The focus has shifted from just anti-sickling properties to address broader issues like vaso-occlusive crisis. Enrollment challenges: Low patient participation in clinical trials slows progress. Data scarcity: Lack of accurate data on prevalence and outcomes makes it difficult to secure funding for research.
Akinsete, 2022 [55]	Commentary	Early Diagnosis and Newborn Screening: Comprehensive care should begin at birth, emphasizing the need for widespread newborn screening programs. Genetic Counseling: Accessible genetic counseling services help at-risk couples make informed decisions and educate families about SCD inheritance patterns. Holistic and Comprehensive Care: A multidisciplinary approach that includes patient education, prophylactic treatment, and prompt care for infections improves survival and quality of life. Stroke Prevention via Transcranial Doppler (TCD) Screening: Routine TCD screening for children aged 2–16 years is critical in preventing stroke. Adapted Healthcare Infrastructure: The WHO AFRO Strategy advocates for improved clinical, laboratory, and imaging facilities adapted to different levels of the health system. Training Healthcare Workers: Continuous training on proper management of SCD and emerging therapies, such as hydroxyurea, is necessary for improving outcomes. NGO Involvement in Care Provision: NGOs play an essential role in raising awareness, providing genetic counseling, stroke prevention programs, and running clinics for affordable care. Advocacy and Political Will: Sustained efforts to implement policies, funding, and legislation for SCD screening and comprehensive care are vital to improving SCD outcomes. International Collaboration: Partnerships that provide access to cutting-edge treatments, like new drugs, stem cell transplantation, and gene therapy, can transform the future of SCD care. Cost Reduction and Universal Health Coverage (UHC): Implementing UHC and reducing treatment costs will improve accessibility to SCD care, particularly for vulnerable populations.	The lessons learned from the management of sickle cell anemia (SCA) in Africa highlight the importance of early diagnosis and comprehensive care in improving survival rates. Newborn screening and genetic counseling have proven effective, though such services are limited to a few centers, leaving many patients undiagnosed until severe symptoms arise. Holistic care, which includes patient education, vaccinations, and malaria prevention, reduces complications, while stroke prevention through transcranial Doppler screening has shown to significantly lower stroke risks in children with SCA. However, barriers such as poor healthcare infrastructure, limited diagnostic capacity, and inadequate training among clinicians hinder effective management. NGOs play a crucial role in providing care, raising awareness, and advocating for SCA policies, but stronger political commitment from African governments is needed to fully integrate SCA control programs into national health systems. Additionally, while global research offers valuable insights, its application in Africa must consider local environmental and health system differences. High treatment costs further exacerbate the challenges, emphasizing the need for affordable healthcare solutions and expanded coverage.
Ally, 2023 [56]	Review	Early Diagnosis: Newborn screening (NBS) programs enable early detection and timely intervention. Examples: Ghana, Nigeria, Tanzania, Zambia. Comprehensive Care: Specialized SCD clinics provide pain management, infection prevention, blood transfusions, and psychosocial support. Examples: Muhimbili National Hospital (Tanzania), National Sickle Cell Centre (Nigeria). Improved Access to Care: National health insurance schemes and government support increase access to affordable treatment, especially in rural areas. Examples: Tanzania, Nigeria. Public Health Initiatives: National strategies prioritize SCD prevention and control. Examples: WHO African Region strategy, National Sickle Cell Control Act (USA). Community Engagement: Public education campaigns to address misconceptions and encourage early diagnosis and treatment adherence. Examples: Sickle Cell Foundation Nigeria, SickleInAfrica consortium. Collaborative Approach: Partnerships between healthcare providers, policymakers, and community organizations help address SCD challenges. Examples: CONSA, PEN-Plus project.	Limited Resources: Lack of funding, infrastructure, and trained professionals can impede effective SCD management. Cultural Barriers: Cultural beliefs impact health-seeking behaviors and treatment adherence, requiring tailored interventions. Data Scarcity: Limited data on SCD prevalence and outcomes hinder research and policy-making. Example: Lack of newborn screening data in many countries. Challenges in Care Delivery: Shortages of diagnostic tests, medications (e.g., hydroxyurea), and specialized care (e.g., TCD machines for stroke screening) limit treatment options. Need for Innovation: Ongoing research into gene therapy and other advanced treatments is crucial to address evolving SCD management challenges.
Arji, 2023 [57]	Review	Comprehensive Care: Holistic care models, including patient education and management of acute manifestations, significantly improve clinical outcomes and survival rates. Disease-Modifying Agents: Hydroxyurea (HU) therapy is highly effective in reducing SCD-related adverse events and should be widely accessible. Newborn Screening: NBS combined with comprehensive primary healthcare is cost-effective and reduces mortality. Nutritional Support: Arginine and omega-3 supplementation can improve clinical outcomes and metabolic health. Pharmacotherapy: Antimalarial drugs and supportive therapies like blood transfusions are crucial for managing SCD, especially in malaria-endemic regions. Patient and Caregiver Education: Organized social care services that provide intensive education and follow-up are essential for improving outcomes.	Resource Allocation: Expanded access to essential therapies like HU and improved healthcare infrastructure are crucial for effective SCD management. Early Diagnosis and Intervention: NBS programs need to be implemented more widely to reduce mortality. Pharmacotherapy Challenges: Drug resistance and variability in efficacy of antimalarial drugs require ongoing research and monitoring. Supportive Care: Ensuring access to blood products and trained personnel for supportive therapies like blood transfusions is essential. Patient Education: Scaling up patient and caregiver education programs is necessary for improving adherence to treatment and overall outcomes.
Oron, 2020 [58]	Commentary	Early Screening and Diagnosis: Newborn screening programs using POCTs like SickleScan™ and HemoTypeSC™ are effective for early detection and intervention. Comprehensive Preventive Care: A package including early screening, penicillin prophylaxis, antimalarial treatments, and vaccinations is essential for reducing mortality. Hydroxyurea Therapy: HU is a highly effective disease-modifying agent that reduces anemia, painful crises, and stroke. Sustainability of Screening Programs: Local government and global health organization support is crucial for long-term success. Community Engagement: Addressing societal attitudes and fostering caregiver acceptance is essential for improving outcomes. Cost-Effectiveness of Comprehensive Care: Investing in comprehensive care for SCD is economically beneficial, with significant savings in healthcare costs and improved quality of life.	Financial Sustainability: Long-term funding from local governments and global health organizations is essential for maintaining screening programs and ensuring access to essential treatments. Community Involvement: Engaging with communities to address cultural beliefs and stigma is crucial for improving healthcare outcomes. Cost–Benefit Analysis: Economic evaluations demonstrate the financial viability of comprehensive SCD care in Africa. Multidisciplinary Approach: Collaborative efforts involving healthcare providers, policymakers, and community organizations are necessary for successful SCD management.

## Data Availability

No new data were created or analyzed in this study. Data sharing is not applicable to this article.

## References

[1] Williams T.N. (2024). The Clinical Epidemiology of Sickle Cell Disease in Sub-Saharan Africa. Sickle Cell Disease in Sub-Saharan Africa.

[2] Dilli P.P., Obeagu E., Tamale A., Ajugwo A., Pius T. (2024). Makeri Update on the practice of premarital screening for sickle cell traits in Africa: A systematic review and meta-analysis. BMC Public Health.

[3] Ojelabi A. (2024). Sickle Cell Disease in Africa. Public Health in Sub-Saharan Africa: Social Epidemiological Perspectives.

[4] WHO. World Health Organization (2021). Sickle Cell Disease. https://www.afro.who.int/health-topics/sickle-cell-disease.

[5] Naik R.P., Haywood J.C. (2015). Sickle cell trait diagnosis: Clinical and social implications. Hematol. 2014 Am. Soc. Hematol. Educ. Program Book.

[6] Gonçalves B.P., Gupta S., Penman B.S. (2016). Sickle haemoglobin, haemoglobin C and malaria mortality feedbacks. Malar. J..

[7] Piel F.B., Patil A.P., Howes R.E., Nyangiri O.A., Gething P.W., Williams T.N., Hay S.I. (2010). Global distribution of the sickle cell gene and geographical confirmation of the malaria hypothesis. Nat. Commun..

[8] Inusa B., Nwankwo K., Azinge-Egbiri N., Bolarinwa B. (2024). Sickle Cell Disease in Sub-Saharan Africa: Biomedical Perspectives.

[9] Munung N.S., Nnodu O.E., Moru P.O., Kalu A.A., Impouma B., Treadwell M.J., Wonkamet A. (2024). Looking ahead: Ethical and social challenges of somatic gene therapy for sickle cell disease in Africa. Gene Ther..

[10] Peter P.D., Makeri D. (2024). Winning the fight against sickle cell disease in Africa: The need to redefine premarital sickle cell trait screening to promote awareness. J. Educ. Health Promot..

[11] Ojelabi A.O. (2018). Quality Of Life and its Predictor Markers in Sickle Cell Disease in Ibadan South West Nigeria.

[12] Lucchesi F., Figueiredo M.S., Mastandrea E.B., Levenson J.L., Smith W.R., Jacinto A.F., Citero W.d.A. (2016). Physicians’ perception of sickle-cell disease pain. J. Natl. Med. Assoc..

[13] Booth C., Inusa B., Obaro S.K. (2010). Infection in sickle cell disease: A review. Int. J. Infect. Dis..

[14] Tluway F., Makani J. (2017). Sickle cell disease in Africa: An overview of the integrated approach to health, research, education and advocacy in Tanzania, 2004–2016. Br. J. Haematol..

[15] Rees D.C., Brousse V.A., Brewin J.N. (2022). Determinants of severity in sickle cell disease. Blood Rev..

[16] Dexter D., McGann P.T. (2023). Hydroxyurea for children with sickle cell disease in sub-Saharan Africa: A summary of the evidence, opportunities, and challenges. Pharmacother. J. Hum. Pharmacol. Drug Ther..

[17] Diop S., Pirenne F. (2021). Transfusion and sickle cell anemia in Africa. Transfus. Clin. Biol..

[18] Smart L.R., Hernandez A.G., Ware R.E. (2018). Sickle cell disease: Translating clinical care to low-resource countries through international research collaborations. Seminars in Hematology.

[19] Therrell B.L., Lloyd-Puryear M.A., Ohene-Frempong K., Ware R.E., Padilla C.D., Ambrose E.E., Barkat A., Ghazal H., Kiyaga C., Mvalo T. (2020). Empowering newborn screening programs in African countries through establishment of an international collaborative effort. J. Community Genet..

[20] Piel F.B., Rees D.C., DeBaun M.R., Nnodu O., Ranque B., Thompson A.A., Ambrose E.E., Andemariam P.B., Colah R., Colombatti R. (2023). Defining global strategies to improve outcomes in sickle cell disease: A Lancet Haematology Commission. Lancet Haematol..

[21] WHO (2024). WHO 2024 Package of Interventions for Sickle Cell Disease Management. https://www.afro.who.int/publications/who-sickle-package-interventions-sickle-cell-disease-management.

[22] Jesson J.K., Lacey F.M. (2006). How to do (or not to do) a critical literature review. Pharm. Educ..

[23] Guindo A., Cisse Z., Keita I., Desmonde S., Sarro Y.D.S., Touré B.A., Baraika M.A., Tessougué O., Guindo P., Coulibaly M. (2024). Potential for a large-scale newborn screening strategy for sickle cell disease in Mali: A comparative diagnostic performance study of two rapid diagnostic tests (SickleScan^®^ and HemotypeSC^®^) on cord blood. Br. J. Haematol..

[24] Datta A., Tsouana E., Ominu-Evbota K., Tuffin N. (2023). 480 Suspected Renal Papillary Necrosis in Children with Sickle Cell Disease.

[25] Adan J.D., Batte A., Namazzi R., Mufumba I., Kazinga C., Mellencamp K.A., Bond C., Opoka R.O., John C.C., Conroy A.L. (2023). Renin as a biomarker of acute kidney Injury and mortality in children with severe malaria or sickle cell disease. Cureus.

[26] Ladu A.I., Jeffery C., Farate A., Farouk A.G., Abba A.M., Adekile A., Bates I. (2023). Determinants of splenic preservation among patients with sickle cell disease in North-Eastern Nigeria. Trop. Med. Health.

[27] Mikobi T.M., Kamuanya N.C., Mikobi E.K.B., Kalela T.I., Akilimali P.Z., Lukusa P.T. (2023). Sickle cell anemia and pregnancy: Profile of hemodynamic changes in sickle cell pregnant women in Kinshasa. EJHaem.

[28] Bello-Manga H., Galadanci A.A., Abdullahi S., Ali S., Jibir B., Gambo S., Gambo S., Haliru L., Jordan L.C., Aliyu M.H. (2020). Low educational level of head of household, as a proxy for poverty, is associated with severe anaemia among children with sickle cell disease living in a low-resource setting: Evidence from the SPRING trial. Br. J. Haematol..

[29] Namugerwa C.H., Gavamukulya Y., Barugahare B.J. (2023). Knowledge and attitude towards sickle cell anemia among care givers of paediatric sickle cell patients at a tertiary hospital in Eastern Uganda: A cross sectional study. BMC Res. Notes.

[30] Olaniyan H.S., Briscoe C., Santos B., Pascoal R., Armando A., McGann P.T. (2021). Comparison of Sickle SCAN and Hemotype SC As Point-of-Care Newborn Screening Diagnostics for Sickle Cell Disease in Luanda, Angola. Blood.

[31] Aimé A.K., Mick S.Y.P., Aimée M.M., Léon T.M., Etienne S.M., Stanis W.O. (2024). Hospital Prevalence of Sickle Cell Disease in Children Under 5 Years of Age in a Region of the Democratic Republic of Congo. Eur. J. Med. Health Res..

[32] Ally M., Mauti G.O. (2023). A Cross-Sectional Study Of The Prevalence Of Sickle Cell Disease Among Children Of Under The Age Of Five Years At Heri Mission Hospital In Buhigwe District–Kigoma. Stud. J. Health Res. Afr..

[33] Ambrose E.E., Smart L.R., Charles M., Hernandez A.G., Latham T., Hokororo A., Latham T., Hokororo A., Beyanga M., Howard T.A. (2020). Surveillance for Sickle Cell Disease.

[34] Khisa T., Omwenga E., Emonyi W. (2021). Sickle cell trait frequencies in blood donors and its effect among recepients in Bungoma County, Kenya. East Afr. Med. J..

[35] Adam M.A., Adam N.K., Mohamed B.A. (2019). Prevalence of sickle cell disease and sickle cell trait among children admitted to Al Fashir Teaching Hospital North Darfur State, Sudan. BMC Res. Notes.

[36] Katamea T., Mukuku O., Mpoy C.W., Mutombo A.K., Luboya O.N., Wembonyama S.O. (2024). Newborn screening for sickle cell disease in Lubumbashi, Democratic Republic of the Congo: An update on the prevalence of the disease. J. Hematol. Allied Sci..

[37] Kasai E.T., Gulbis B., Ntukamunda J.K., Bours V., Batina Agasa S., Marini Djang’eing’a R., Boemer F., Katenga Bosunga G., Ngbonda Dauly N., Sokoni Vutseme L.J. (2023). Newborn screening for sickle cell disease in Kisangani, Democratic Republic of the Congo: An update. Hematology.

[38] Mano R.M., Kuona P., Misihairabgwi J.M. (2024). Determination of birth prevalence of sickle cell disease using point of care test HemotypeSC™ at Rundu Hospital, Namibia. BMC Pediatr..

[39] Namukasa S., Maina R., Nakaziba S., Among G., Asasira L., Mayambala P., Atukwatse J., Namuguzi M., Ahmed M. (2024). Sarki Prevalence of sickle cell trait and needs assessment for uptake of sickle cell screening among secondary school students in Kampala City, Uganda. PLoS ONE.

[40] Chimbatata C.S., Chisale M.R., Kayira A.B., Sinyiza F.W., Mbakaya B.C., Kaseka P.U., Kamudumuli P., Wu T.S.J. (2021). Paediatric sickle cell disease at a tertiary hospital in Malawi: A retrospective cross-sectional study. BMJ Paediatr. Open.

[41] Oppong M., Lamptey H., Kyei-Baafour E., Aculley B., Ofori E.A., Tornyigah B., Kweku M., Ofori M.F. (2020). Prevalence of sickle cell disorders and malaria infection in children aged 1–12 years in the Volta Region, Ghana: A community-based study. Malar. J..

[42] Sawe H.R., Reynolds T.A., Mfinanga J.A., Runyon M.S., Murray B.L., Wallis L.A., Makani J. (2018). The clinical presentation, utilization, and outcome of individuals with sickle cell anaemia presenting to urban emergency department of a tertiary hospital in Tanzania. BMC Hematol..

[43] Sa A., Bg O. (2020). Book Chapter: Clinical Features of Sickle Cell Disease in Children. Sick. Cell Dis. Lab. Clin. Practice..

[44] Isa H., Adegoke S., Madu A., Hassan A.-A., Ohiaeri C., Chianumba R., Brown B., Okocha E., Ugwu N., Diaku-Akinwumi I. (2020). Sickle cell disease clinical phenotypes in Nigeria: A preliminary analysis of the Sickle Pan Africa Research Consortium Nigeria database. Blood Cells Mol. Dis..

[45] Adebayo O.C., Betukumesu D.K., Nkoy A.B., Adesoji O.M., Ekulu P.M., Van den Heuvel L.P., Levtchenko E.N., Labarque V. (2022). Clinical and genetic factors are associated with kidney complications in African children with sickle cell anaemia. Br. J. Haematol..

[46] Mbayabo G., Ngole M., Lumbala P.K., Lumaka A., Race V., Matthijs G., Mikobi T.M., Devriendt K., Van Geet C., Lukusa P.T. (2023). Clinical and biological profile of Sickle Cell Anemia children in a rural area in Central Africa. Hematology.

[47] Lumbala P.K., Mbayabo G., Ngole M.N., Lumaka A., Race V., Matthijs G., Madu K. (2022). Clinical and laboratory characterization of adult sickle cell anemia patients in Kinshasa. PLoS ONE.

[48] Duru A., Madu A.J., Okoye H., Nonyelu C., Obodo O., Okereke K., Madu K. (2021). Variations and characteristics of the various clinical phenotypes in a cohort of Nigerian sickle cell patients. Hematology.

[49] Sahli A., Ouali F., Dabboubi R., Fredj S.H., Meddeb N., Mzoughi N., Messaoud T. (2023). Epidemiological and clinical characteristics of 66 Tunisian Sickle cell syndrome patients. Afr. Health Sci..

[50] Hassan I.I., Anazodo M., Lawal A.A., Bello S.O. (2022). Clinical Profile of Children with Sickle Cell Anaemia in Nasarawa State, Nigeria: A Five–Year Review. J. Med. Women’s Assoc. Niger..

[51] Esoh K., Wonkam-Tingang E., Wonkam A. (2021). Sickle cell disease in sub-Saharan Africa: Transferable strategies for prevention and care. Lancet Haematol..

[52] Coetzee W., Khumalo R., Le Roux B., Van Wyk E. (2022). Sickle Cell Disease: Causes, Symptoms, and Treatment. Fusion Multidiscip. Res. Int. J..

[53] Egesa W.I., Nakalema G., Waibi W.M., Turyasiima M., Amuje E., Kiconco G., Odoch S., Kumbakulu P.K., Abdirashid S., Asiimwe D. (2022). Sickle Cell Disease in Children and Adolescents: A Review of the Historical, Clinical, and Public Health Perspective of Sub-Saharan Africa and Beyond. Int. J. Pediatr..

[54] Olukorede D.E., Farayola O.R., Badmus B.M., Adebisi Y.A. (2022). Sickle cell anaemia: The need for increased drug development in Africa. Ann. Public Health Issues.

[55] Akinsete A. (2022). Treatment of sickle cell disease in sub-Saharan Africa: We have come a long way, but still have far to go. J. Glob. Med..

[56] Ally M., Balandya E. (2023). Current Challenges and New Approaches to Implementing Optimal Management of Sickle Cell Disease in Sub-Saharan Africa Seminars in Hematology.

[57] Arji E.E., Eze U.J., Ezenwaka G.O., Kennedy N. (2023). Evidence-based interventions for reducing sickle cell disease-associated morbidity and mortality in sub-Saharan Africa: A scoping review. SAGE Open Med..

[58] Oron A.P., Chao D.L., Ezeanolue E.E., Ezenwa L.N., Piel F.B., Ojogun O.T., Uyoga S., Williams T.N., Nnodu O.E. (2020). Caring for Africa’s sickle cell children: Will we rise to the challenge?. BMC Med..

[59] Adigwe O.P., Onoja S.O., Onavbavba G. (2023). A Critical Review of Sickle Cell Disease Burden and Challenges in Sub-Saharan Africa. J. Blood Med..

[60] Thomson A.M., McHugh T.A., Oron A.P., Teply C., Lonberg N., Tella V.V., Wilner L.B., Fuller K., Hagins H., Aboagye R.G. (2023). Global, regional, and national prevalence and mortality burden of sickle cell disease, 2000–2021: A systematic analysis from the Global Burden of Disease Study 2021. Lancet Haematol..

[61] Meier E.R., Miller J.L. (2012). Sickle cell disease in children. Drugs.

[62] Franco R.S., Yasin Z., Palascak M.B., Ciraolo P., Joiner C.H., Rucknagel D.L. (2006). The effect of fetal hemoglobin on the survival characteristics of sickle cells. Blood.

[63] Kanter J., Kruse-Jarres R. (2013). Management of sickle cell disease from childhood through adulthood. Blood Rev..

[64] Ceglie G., Di Mauro M., Tarissi De Jacobis I., De Gennaro F., Quaranta M., Baronci C., Villani A., Palumbo G. (2019). Gender-related differences in sickle cell disease in a pediatric cohort: A single-center retrospective study. Front. Mol. Biosci..

[65] El-Haj N., Hoppe C.C. (2018). Newborn Screening for SCD in the USA and Canada. Int. J. Neonatal Screen.

[66] Nkya S., Njiro B.J., Ngowi D., Solomon D., Kaywanger F., Nyangasa S., Ndoje G., Marco E., Moses M., Makani J. (2022). Building research capacity for sickle cell disease in Africa: Lessons and challenges from establishing a birth cohort in Tanzania. Front. Pediatr..

[67] Obeagu E.I., Adias T.C. (2024). Global Partnerships: Collaborative Efforts for International Sickle Cell Disease Education. Int. J. Curr. Res. Chem. Pharm. Sci..

